# Association between diet and physical activity and sedentary behaviours in 9–10-year-old British White children

**DOI:** 10.1016/j.puhe.2012.12.006

**Published:** 2013-03

**Authors:** P.A.J. Vissers, A.P. Jones, E.M.F. van Sluijs, A. Jennings, A. Welch, A. Cassidy, S.J. Griffin

**Affiliations:** a School of Environmental Sciences, University of East Anglia, Norwich NR4 7TJ, UK; b MRC Epidemiology Unit, Institute of Public Health, Cambridge, UK; c Norwich Medical School, University of East Anglia, Norwich, UK

**Keywords:** Diet, Physical activity, Children, Sedentary behaviour

## Abstract

**Objectives:**

To examine the association between diet and physical activity and sedentary behaviours in 9–10-year-old children.

**Study design:**

A cross-sectional study using data from the SPEEDY (Sport, Physical activity and Eating behaviour: Environmental Determinants in Young People) study undertaken in Norfolk, UK.

**Methods:**

Data from 4-day food diaries and 7 days of accelerometery were matched on concurrent days. Time spent in moderate-to-vigorous physical activity (MVPA), time spent in sedentary behaviour and various measures of dietary intake were collected. Covariates included age, sex, weight status, family socio-economic status, and energy intake reporting quality. Multivariable regression models, adjusted for clustering of children by school and stratified by sex, were fitted to examine the associations between dietary measures and physical activity and sedentary outcomes.

**Results:**

In total, 1317 children (584 boys and 733 girls) provided concurrent data. Boys in the highest quartile of energy percentage from protein spent approximately 6 min [95% confidence interval (CI) 0–12] less in MVPA compared with boys in the lowest quartile. Those in the highest quartiles of fruit and vegetable intake and fruit juice intake had respective average activity counts per minute that were 56 above (95% CI 8–105) and 48 below (95% CI 2–95) those in the lowest quartiles, whilst those in the highest quartile of fizzy drink consumption spent approximately 7 min (95% CI 2–13) more in MVPA and approximately 14 min (95% CI 5–24 min) less in sedentary behaviour. Boys in the highest quartile of savoury snack consumption spent approximately 8 min (95% CI 2–13 min) more in MVPA per day, and approximately 12 min (95% CI 2–23) less in sedentary behaviour. No significant associations were apparent among girls.

**Conclusions:**

Few associations were detected, and the directions of those that were apparent were mainly counterintuitive. The extent to which this reflects a true lack of association or is associated with the measurement methods used for diet and physical activity needs further investigation.

## Introduction

The prevalence of overweight and obesity in children is rising sharply.^1–3^ For example, the prevalence in England increased from 11% in 1995 to 17% in 2008 among 2–15-year-old boys, and from 12% to 15% in 2–15-year-old girls.^4^ This is of concern as obese children are more likely to develop psychological and physiological problems. They are known to be more likely to have lower self-esteem and more behavioural problems than non-obese children.^5^ Furthermore, several cardiovascular risk factors, such as hypertension, dyslipidaemia, hyperinsulinaemia and insulin resistance, are associated with childhood obesity.^6^ A further concern is that childhood obesity tracks into adulthood,^7^ which presents a risk for a range of chronic conditions including cardiovascular disease, musculoskeletal disorders and type 2 diabetes.^8^ Although the precise mechanisms of childhood obesity remain unclear, the presence of energy imbalance (higher energy intake than expenditure) is pivotal.^9^

Several dietary behaviours have been associated with overweight in children, with total energy intake,^10^ percentage energy from fat, and energy density^11^ all identified as risks. Conversely, higher fruit and vegetable intake has been found to be protective against weight gain.^12^ Worryingly, the British National Diet and Nutrition Survey found that average intakes of saturated fat, salt and non-milk intrinsic sugars are above recommended levels amongst young people, whilst fruit, vegetable and fibre intakes are below recommended levels.^13^ In addition, energy expenditure appears low, with physical activity levels below recommended levels in British children and adolescents.^14^ One-third of boys and one-third to one-half of girls report activity levels that may compromise their health.^15^

There are indications that diet may be correlated with physical activity and sedentary behaviours, but associations are not well understood in children. In adults, higher physical activity has been associated with higher consumption of fruit, fruit juices and vegetables,^16–19^ whilst high consumption of energy from fat has been associated with lower activity levels.^16,17,19,20^ Evidence in young people often comes from studies focusing on sedentary behaviour, including television and computer use. In 16–20-year-old English adolescents, it was found that more time spent watching films at home at the weekend was related to higher total energy intake, higher fat intake, higher percentage energy intake from fat and lower carbohydrate intake.^21^ A multicountry study found that children who watched more television were more likely to consume more sweets and soft drinks, and less fruit and vegetables.^22^ Television and computer use has also been inversely associated with fruit and vegetable intake in 5–17-year-old children in Scotland,^23^ and a recent systematic review suggested that television viewing is consistently associated with lower fruit and vegetable consumption, and higher energy-dense snack consumption in children.^24^

Two studies investigated associations with physical activity. The first showed a negative association with fat intake and a positive association with carbohydrate intake in 8–10-year-old African–American girls.^25^ A second study in 10–11-year-old children in England reported a negative association between physical activity and fat intake, and a positive association with total energy and carbohydrate intake amongst boys. In girls, fruit and vegetable intake was consistently positively associated with physical activity.^26^ Taken together, these studies suggest some association between dietary intakes and physical activity, but this association may be moderated by gender.

The evidence to date on the relationship between diet and physical activity in children has a number of limitations. Many previous studies^21–23^ have assessed dietary intakes using food frequency questionnaires, and assessed physical activity with self-reported questionnaires, rather than use food diaries to assess dietary intakes and measure physical activity objectively by accelerometry.^27,28^ Just one study, that of Jago *et al.*,^26^ used both those methods, although the diet and physical activity data were recorded 12 months apart which may attenuate the strength of associations present.

The objective of this study was to extend the evidence base on the association between diet and physical activity, including sedentary behaviour, by analysing data collected amongst a well-characterised sample of 9–10-year-old British children using validated dietary and physical activity instruments. A 4-day food diary was used to assess dietary intake, and physical activity measurements were based on accelerometery.

## Methods

### Study sample and analytical design

This cross-sectional analysis used data from the SPEEDY (Sport, Physical activity and Eating behaviour: Environmental Determinants in Young People) study, undertaken to examine physical activity and dietary behaviours in a population-based sample of Year 5 (aged 9–10 years) children in Norfolk, England. A detailed overview of the sampling and data collection has been published elsewhere.^29^ In brief, the study sample consisted of 2064 children attending 92 schools between April and July 2007. Each school was visited by a team of research assistants who distributed a range of items including a 4-day food diary, an accelerometer for the measurement of physical activity, and a questionnaire for the parent or carer of each child. A range of anthropometric measurements was also taken during the school visit.

Data from 4-day food diaries were matched with data on physical activity recorded during the same period in order to assess the relationship between eating and physical activity behaviours. Data collection and variable generation is described below.

### Physical activity

Free-living physical activity was assessed using an accelerometer, the Actigraph GT1M activity monitor (Actigraph LCC, Pensacola, FL, USA) which has been validated in 9-year-old children.^30^ The children wore the monitor for 7 days on their right hip during waking hours, except whilst in water. For the purposes of this analysis, only accelerometery recorded during the period of food diary completion was utilised. Children kept a diary to record when and why they had removed the monitor. Activity data were stored at 5-s intervals; when 10 min of no motion was measured, it was classed as non-worn time, according to the protocol used by others.^31–33^ The outcome variables were activity counts per minute (CPM) between 6 am and 11 pm, sedentary time and moderate-to-vigorous physical activity (MVPA). CPM measures the amount of acceleration recorded by the Actigraph, and can therefore be used to infer the intensity of physical activity. Sedentary time was defined as accelerometer recordings between 0 and 100 CPM, and time spent in MVPA was defined as accelerometer recordings above 2000 CPM. This upper threshold corresponds to a walking pace of approximately 4 km/h in children, and has previously been applied successfully in this age group to study associations between the intensity of physical activity and metabolic outcomes.^34^ Children were excluded from this analysis if they did not record valid data for at least 500 min/day on at least 3 days. The use of 500 min to define a valid day has been shown to provide reliable estimates of physical activity in children.^32,35^ Children who reported physical activity and dietary intake on just 1 or 2 matched days were excluded from the analysis, as recorded values for such short periods may be strongly influenced by short-term abnormal physical activity or dietary behaviours.

### Dietary intake

Dietary intake was assessed using a 4-day food diary. Assessment days were consecutive and included two weekdays and two weekend days. With assistance from their parents or carers, children recorded all foods and drinks consumed and estimated the portion size of each item. The weights of the portions were then approximated using published values for children.^36–38^ Mean nutrient intakes were estimated using WISP Nutritional Analysis Software Version 3.0 (Tinuviel Software, Warrington, UK) using nutrient values from McCance and Widdowson.^39^ Nutrient intakes were checked for outliers prior to analysis. Where outliers were found, individual diaries were examined for data entry errors. If errors existed, the relevant amendments were made and the database was updated accordingly. As this study focused on the association between diet and physical activity, dietary outcome variables that were found to be related to overweight or obesity in previous studies were chosen.^10–12,40,41^

The following measures were studied, for which daily values were aggregated and averaged over the 4 days: energy intake (kcal); energy percentage from fat, saturated fat, carbohydrate and protein; and energy density. The measure of energy density was calculated by dividing total energy intake from all solid foods (kJ) by their total weight (g). Additionally, eight food groups were included in the analysis: fruit and vegetable intake (excluding potatoes/fries and baked beans), natural fruit juices, fizzy drinks (soda), squash drinks (non-carbonated fruit drinks), savoury snacks (potato and non-potato based), sweet snacks (including cakes, biscuits, pastry, buns and puddings), chocolate/sweets and fries. All food groups were relative to total energy intake in megajoules (MJ). As their dietary composition was significantly different from those who completed the full 4 days (results not shown), children who reported less than 4 days of dietary data were excluded from the analysis.

To assess potential dietary under-reporting, the ratio of reported energy intake to estimated energy requirement was calculated, as has been practised elsewhere.^11,42^ Estimated energy requirement was calculated using the methodology of the joint World Health Organization (WHO), the Food and Agriculture Organization (FAO) and United Nations University (UNU) Expert Consultation Report on Human Energy Requirements.^43^ For the SPEEDY study, the 95% confidence interval (CI) for energy intake:estimated energy requirement was 0.71–1.30%, and therefore reports of 4-day mean energy intake within the range of 71–130% of energy intake:estimated energy requirement were considered to be in the range of normal measurement, suggesting that 77% of the children had plausible reports of energy intake.^44^ As it has been shown that the exclusion of children who under or over report their dietary intake can distort dietary estimates,^45^ energy reporting quality was included as a covariate in statistical models.

### Anthropometry

The fat mass index (FMI) of each child was analysed as a covariate. The measure was obtained from anthropometric measurements taken during the school visit. Height was measured to the nearest millimetre using a portable Leicester height measure (Birmingham, United Kingdom). A non-segmental bio-impedance scale (Tanita TBF-300A, Tokyo, Japan) was used to assess impedance. FMI was derived from the impedance value using previous validated and published equations.^46^ FMI [fat mass (kg)/height (m)^2^] was then calculated for each child. FMI was chosen over the more commonly used body mass index (BMI) as it has been shown to be more closely correlated with measures of physical activity in this sample.^47^

### Statistical analysis

Physical activity data from the accelerometer were matched to the days each child provided food diary data. Matched data were aggregated and studied as daily means over the 3- or 4-day study period.

The potential moderating effects of gender were tested using interaction terms, and significant differences between boys and girls were observed at the *P* < 0.1 level in at least one of the outcomes for three of the exposures considered. As previous studies had also found differences by gender, analyses were stratified by gender. The physical activity outcomes were entered as dependent variables in regression models, whilst the averaged dietary metrics were entered as independent variables. Separate models were fitted for each physical activity and dietary measure.

As the sampling strategy for the SPEEDY study was based around schools, there was the potential for school-level clustering of outcomes. Consequently, multilevel (random coefficient) models were specified, with children (level 1) nested within schools (level 2). The models were fitted using MLwiN Version 2.18, Bristol, United Kingdom.^48^ Total valid wear time registered by the accelerometer was included in the models for sedentary behaviour and MVPA to account for any artefacts associated with wear time. Child age, FMI and the ratio of reported to expected energy intake were included as covariates in all the models. Socio-economic status, measured in this study according to the highest educational attainment of the parent or guardian of the child, was not found to be associated with either set of outcomes, and thus was not included as a covariate.

In the regression models, the coefficients are reported as predicted means of the physical activity outcomes, holding the other covariates to their means. The dietary measures were modelled as quartiles, and parameter estimates were computed and compared for each. To test for the strength of any linear trends across quartiles, the quartiles were modelled as a continuous variable and statistical significance was recorded. A *P*-value of <0.05 was regarded as significant in all analyses.

## Results

### Baseline characteristics of the study population

Out of 2064 children participating in the SPEEDY study, 1974 (96%) wore an accelerometer and 1868 (95%) provided valid physical activity data. In total, 1859 (90%) children completed a food diary, of which 1718 (92%) provided valid dietary data on all 4 days of assessment. In total, 1317 (64%) children (584 boys and 733 girls) provided dietary and physical activity data on at least 3 concurrent days, and were included in this analysis. The included children did not differ in either age {10.2 years [standard deviation (SD) 0.3] vs 10.3 years (SD 0.3); *P* = 0.10}, sex (44.3% vs 45.8% boys; *P* = 0.53) or FMI [5.8 kg/m^2^ (SD 2.6) vs 5.8 kg/m^2^ (SD 2.6); *P* = 1.00] compared with the excluded children.

Characteristics of the included sample are shown in Table 1. Boys had a significantly lower FMI compared with girls, and also spent less time in sedentary behaviour, more time in MVPA and had a higher mean CPM. Furthermore, boys had a higher total energy intake, higher energy percentage from protein, higher fizzy drink and squash intake, and a lower fruit and vegetable intake.

### Associations between diet and physical activity

The models showing adjusted associations between the physical activity and dietary outcomes are shown in Tables 2 and 3 for boys and girls, respectively. Few significant associations are apparent based on tests for trend. Boys in the highest quartile of energy percentage from protein spent approximately 6 min (95% CI 0–12) less in MPVA. Those in the highest quartile of fruit and vegetable intake and fruit juice intake had respective average mean CPMs that were 56 above (95% CI 8–105) and 46 below (95% CI 2–95) those in the lowest quartile. Boys in the highest quartile of fizzy drink consumption spent approximately 7 min (95% CI 2–13) more in MVPA and approximately 14 min (95% CI 5–24) less in sedentary behaviour compared with those in the lowest quartile. Increased savoury snack intake was associated with lower levels of sedentary behaviour and higher MVPA. Boys in the highest quartile of savoury snack consumption spent approximately 8 min (95% CI 2–13) more in MVPA and approximately 12 min (95% CI 2–23) less in sedentary behaviour compared with those in the lowest quartile. In girls, no significant associations between any of the physical activity outcomes and the dietary measures were apparent, and effect sizes were generally small.

## Discussion

In this study, no clear relationship was detected between physical activity and dietary patterns in 9–10-year-old British children. In boys, some associations were in the direction expected. For example, higher fruit and vegetable consumption was associated with higher mean CPM. However, many of the directions of association were counter-intuitive, such as fizzy drink and savoury snack intakes being positively associated with MVPA. In girls, no significant associations were found. All associations were relatively weak, and differed by less than 14 min of MVPA or sedentary behaviour when comparing the highest and lowest quartiles. The one previous study conducted in a similarly aged English population also failed to find strong associations, but did find a negative association between energy percentage from fat and physical activity in boys. In that study, fruit and vegetable intake was positively associated with physical activity in girls, as found in boys in the present study. However, the dietary and physical activity measures were taken 12 months apart.^26^

It is not known why the associations detected in this study were only observed in boys. Boys were not more variable than girls in their physical activity or dietary intakes, so the difference is not likely to be associated with more heterogeneity in the sample of boys. The fact that boys were generally more physically active than girls may mean that dietary quality is more important for them, although given that a large number of tests were undertaken and some of the associations that were observed in boys were in a counter-intuitive direction, the difference between boys and girls in the number of associations detected may simply be due to chance.

The paucity of associations observed in this study could arise from a number of reasons. The authors believe that the use of food diaries and accelerometers in order to measure dietary and physical activity behaviours is a methodological advance over much of the previous literature. It may be that the improved precision of outcome and exposure measurement provided by these methods lessens the influence of poorly controlled confounders in the statistical models. If no true relationship exists, this could explain the lack of associations compared with prior work. The food frequency and activity questionnaires that others have used to assess dietary and physical activity behaviours are likely to measure habitual behaviours more strongly than the more time-specific measures employed in the present study. As both dietary and physical activity behaviours are habitually patterned, it may be that these habitual components of behaviour, representing general dietary quality and physical activity participation, are more dominant than variations in the two behaviours over a short time period.

The strengths of this study include its large population-based study sample and valid assessment of dietary intake and physical activity. As dietary and physical activity measures were conducted in the same time period, it was possible to record both behaviours concurrently. Dietary intake was assessed with a 4-day food diary, which has been shown to exhibit better agreement between observed and reported dietary intake compared with 24-h recall and 5-day food frequency questionnaires in 9–10-year-old girls.^49^ Furthermore, an accelerometer comparable to that used in the present study has been validated against energy expenditure estimated by the doubly-labelled water method in 9-year-old children.^30^ All measurements were conducted during the summer term, which reduces influences of seasonality on physical activity measurements.

In terms of weaknesses, it is possible that the nature of the food diary could have led to some misclassification of intakes. There is evidence that adults completing diaries report lower intakes of fat and sugar and perceived unhealthy foods, whilst over-reporting intakes of healthy foods such as fruit, vegetables and fish.^50^ The same may be true in children. It has also been suggested that the self-reported nature of dietary intake assessment may alter food intakes when participants eat certain foods for ease of completion or because they are perceived to be healthy.^51^ The cross-sectional study design means that no causal relationships can be established from observed associations. Furthermore, a large number of associations were analysed, and the few that were detected may be due to chance. For sample size purposes, dietary records from weekdays and weekend days were combined in order to produce an average for each child, although there is evidence that both dietary and physical activity patterns of children can differ between school days and weekend days.^52,53^ Nevertheless, the authors investigated if this might affect the findings by fitting separate models for weekday and weekend physical activity (results not presented), and found no substantially different associations to those presented.

FMI was used rather than BMI as FMI provides a direct measure of adiposity. However, measurements were taken whilst the children were at school, and the protocol used did not allow their hydration status to be controlled; this may have affected the values obtained. Nevertheless, the correlation of FMI and BMI within the sample was very high (*r* = 0.97), so this is unlikely to have influenced the findings. For the measures of dietary intake, food intake weights were divided by overall energy intake, rather than model absolute intakes, as this provides a measure of diet quality that is not influenced by differences in overall quantities of food consumed between the children due to variations in energy requirements. However, the analysis was also performed by replacing these contributions with absolute intakes, and the findings were unchanged (results available from corresponding author on request). A final limitation is that the participating children were all recruited from Norfolk. Whilst the environment of the county is varied with both urban and rural areas, Norfolk is more affluent than the national average and has a low percentage non-White population. A higher proportion of girls than boys participated in the SPEEDY study, and only a small proportion of children were from a non-White ethnic background (3.8%).^29^ Hence the findings may not be generalisable to all British children.

In conclusion, there was no clear association between diet and physical activity in this sample of 9–10-year-old British children. The contrast between these null findings and those reported elsewhere in the literature may arise from the fact that associations were examined over a short period rather than habitually in the present study. The extent of dietary reporting bias in children and the implications of using food frequency questionnaires rather than food diaries warrant further investigation.

## Figures and Tables

**Table 1:** Descriptive statistics of the study population reported as mean (standard deviation) unless otherwise stated.

	Boys (*n* = 584)	Girls (*n* = 733)	Total (*n* = 1317)
*Personal characteristics*			
Age (years)	10.2 (0.3)	10.3 (0.3)	10.2 (0.3)
Fat mass index (kg/m^2^)	5.1 (2.3)^b^	6.4 (2.6)	5.8 (2.6)
*Physical activity*			
Sedentary behaviour (min/day)	465.6 (66.5)^a^	473.5 (63.6)	470.0 (65.0)
MVPA (min/day)	84.6 (26.6)^b^	67.4 (21.2)	75.0 (25.2)
Counts per minute	691.2 (220.3)^b^	619.0 (218.8)	651.0 (222.3)
*Diet*			
Energy (MJ)	7.6 (1.5)^b^	7.2 (1.4)	7.4 (1.5)
% Energy from total fat	36.6 (4.6)	37.1 (4.5)	36.9 (4.5)
% Energy from saturated fat	13.8 (2.7)	13.9 (2.6)	13.8 (2.6)
% Energy from protein	14.5 (2.2)^a^	14.2 (2.3)	14.3 (2.3)
% Energy from carbohydrates	48.9 (5.1)	48.7 (4.8)	48.8 (4.9)
Energy density (kJ/g)	6.2 (1.5)	6.3 (1.5)	6.3 (1.5)
Fruit and vegetable intake (g/day)	178.9 (112.8)^a^	196.6 (118.6)	188.7 (116.3)
Fruit juice intake (g/day)	144.3 (154.4)	139.8 (151.4)	141.8 (152.7)
Fizzy drink intake (g/day)	102 (133.4)^b^	68.8 (103.9)	83.6 (119.0)
Squash intake (g/day)	204.6 (245.7)^a^	169.3 (207.6)	184.9 (225.9)
Savoury snack intake (g/day)	12.1 (12.7)	13.3 (11.5)	12.8 (12.0)
Sweet snack intake (g/day)	80.4 (53.5)	79.2 (54.4)	79.7 (54.0)
Chocolate/sweets intake (g/day)	21 (20.5)	20.9 (18.8)	21 (19.6)
Fries intake (g/day)	31.8 (32.8)	32.5 (32.1)	32.2 (32.4)

MVPA, moderate-to-vigorous physical activity.

a Significantly different from girls: *P* < 0.05.

b Significantly different from girls: *P* < 0.01.

**Table 2:** Estimated means and 95% confidence intervals (CI) of physical activity outcomes by quartiles of dietary variables for boys, holding other covariates to their means.

	Sedentary behaviour^a^ (min/day)		Moderate-to-vigorous physical activity^a^ (min/day)		Activity counts per min^b^ (min/day)	
Mean	95% CI	Mean	95% CI	Mean	95% CI
Energy (kcal)						
1. ≤1532.87	463.8	(449.8–477.3)	86.4	(78.9–94.3)	704.6	(640.4–768.3)
2. 1532.88–1796.53	463.7	(454.6–472.1)	85.1	(80.3–89.6)	677.9	(638.5–717.7)
3. 1796.54–2065.20	472.5	(464.5–481.0)	80.8	(76.2–85.2)	669.7	(630.9–708.0)
4. ≥2065.21	461.2	(447.1–475.3)	86.4	(78.6–94.3)	708.3	(641.3–781.0)
En% fat						
1. ≤33.87	465.1	(457.4–473.2)	85.3	(80.9–89.3)	688.9	(653.5–724.2)
2. 33.88–36.74	463.7	(456.1–471.6)	84.7	(80.6–88.8)	685.5	(652.3–720.3)
3. 36.75–39.60	460.6	(452.9–468.7)	87.6	(83.4–91.7)	718.6	(684.1–754.0)
4. ≥39.61	470.8	(463.0–478.5)	81.1	(77.2–85.2)	667.3	(630.7–701.0)
En% saturated fat						
1. ≤11.75	466.8	(459.2–475.1)	83.6	(79.4–87.7)	673.8	(638.7–710.6)
2. 11.76–13.69	469.3	(461.5–477.1)	83.5	(79.4–87.5)	689.7	(656.3–725.7)
3. 13.70–15.69	457.9	(450.4–465.7)	88.7	(84.6–93.1)	704.6	(669.2–741.7)
4. ≥15.7	466.3	(458.6–474.0)	83.1	(79.0–87.2)	692.3	(658.1–727.4)
En% protein						
1. ≤12.93	459.9	(451.8–467.4)	88.1	(83.9–92.4)	704.6	(669.5–740.4)
2. 12.94–14.33	464.2	(456.1–472.1)	84.9	(80.8–88.7)	691.1	(656.1–727.2)
3. 14.34–15.81	466.9	(459.0–474.9)	83.9	(79.7–87.8)	684.8	(650.7–720.7)
4. ≥15.82	469.5	(461.6–477.7)	81.8	(77.8–86.0)^c^	681.2	(646.0–716.4)
En% carbohydrates						
1. ≤45.53	469.0	(461.5–476.9)	82.2	(78.1–86.3)	677.7	(642.9–710.8)
2. 45.54–49.02	464.6	(457.1–472.4)	85.5	(81.5–89.5)	697.1	(664.2–733.8)
3. 49.03–52.32	462.9	(455.4–470.9)	86.2	(82.4–90.3)	706.1	(671.8–740.8)
4. ≥52.33	463.9	(456.0–472.2)	84.8	(80.7–88.8)	678.0	(643.3–713.0)
Energy density (kJ/g)						
1. ≤5.15	466.9	(458.9–474.7)	86.7	(82.5–90.7)	703.5	(668.5–735.7)
2. 5.16–6.08	467.5	(459.7–475.6)	81.5	(77.4–85.5)	672.4	(635.7–707.8)
3. 6.09–7.18	462.0	(454.0–469.6)	87.5	(83.2–91.6)	699.0	(664.5–736.6)
4. ≥7.19	464.5	(456.9–472.4)	83.0	(79.0–87.3)	687.1	(652.0–722.5)
Fruit and vegetable (g/MJ)						
1. ≤13.40	468.2	(460.6–475.6)	82.5	(78.4–86.7)	665.1	(631.6–701.2)
2. 13.41–21.45	466.8	(459.3–475.0)	84.0	(79.7–88.2)	693.8	(656.1–726.8)
3. 21.46–32.10	462.0	(454.3–469.8)	84.5	(80.1–88.6)	681.0	(643.9–715.0)
4. ≥32.11	463.7	(455.9–472.1)	87.6	(83.7–92.0)	722.4	(688.2–755.5)^c^
Fruit juices (g/MJ)						
1. ≤0	462.6	(455.0–469.7)	86.0	(82.0–89.8)	704.1	(671.4–736.1)
2. 0.01–13.41	463.1	(454.9–471.8)	85.3	(81.2–89.9)	720.0	(681.8–758.8)
3. 13.42–29.55	468.2	(459.8–475.5)	83.9	(79.9–88.1)	680.3	(646.4–716.2)
4. ≥29.56	466.2	(458.4–473.5)	83.2	(79.0–87.3)	658.1	(624.3–692.3)^c^
Fizzy drinks (g/MJ)						
1. ≤0	471.7	(465.3–477.9)	81.5	(78.6–84.7)	671.5	(645.1–698.2)
2. 0.01–8.15	447.1	(433.8–460.4)	93.1	(85.7–100.4)	744.9	(682.0–809.9)
3. 8.16–20.40	466.8	(459.2–474.3)	82.8	(78.8–86.8)	669.4	(634.2–703.5)
4. ≥20.41	457.9	(450.5–465.3)^c^	89.1	(85.1–93.2)^c^	727.9	(693.9–762.3)
Squash (g/MJ)						
1. ≤0	464.2	(456.7–471.2)	83.9	(80.1–87.4)	669.0	(637.8–702.0)
2. 0.01–17.11	461.0	(452.1–469.5)	86.3	(81.8–90.8)	722.5	(683.3–765.0)
3. 17.12–39.09	466.4	(458.5–474.6)	83.8	(79.4–87.9)	679.9	(643.6–715.7)
4. ≥39.1	467.8	(460.1–475.8)	85.2	(80.8–89.4)	700.8	(665.4–736.9)
Savoury snacks (g/MJ)						
1. ≤0.58	470.9	(463.0–478.8)	81.7	(77.4–85.8)	669.9	(635.0–705.1)
2. 0.59–1.28	466.3	(459.0–474.2)	82.7	(78.4–86.8)	684.0	(648.5–719.5)
3. 1.29–2.37	464.5	(456.8–472.5)	85.1	(81.2–89.1)	688.5	(655.9–725.4)
4. ≥2.38	458.5	(450.6–466.3)^c^	89.2	(85.0–93.3)^c^	718.6	(683.1–753.4)
Sweet snacks (g/MJ)						
1. ≤5.98	470.6	(462.7–478.6)	82.8	(78.7–86.8)	679.4	(642.6–712.8)
2. 5.99–9.70	460.4	(452.7–468.3)	87.1	(83.0–91.0)	713.0	(678.5–747.6)
3. 9.71–13.88	465.0	(457.3–472.8)	84.6	(80.4–88.6)	679.1	(642.4–715.4)
4. ≥13.89	464.5	(456.9–472.2)	84.2	(80.2–88.3)	691.3	(653.8–726.4)
Chocolate/sweets (g/MJ)						
1. ≤ 0.90	464.8	(457.4–472.5)	84.7	(80.6–88.9)	692.6	(657.0–729.2)
2. 0.91–2.15	467.5	(459.9–475.7)	82.9	(78.6–86.9)	667.1	(631.3–701.8)
3. 2.16–3.89	462.7	(455.2–470.5)	87.0	(83.0–91.0)	720.5	(685.6–755.4)
4. ≥3.9	465.1	(457.1–472.9)	84.0	(80.0–88.1)	680.9	(645.4–715.1)
Fries (g/MJ)						
1. ≤0	467.0	(460.6–473.3)	85.0	(81.6–88.5)	691.0	(661.6–720.4)
2. 0.01–3.34	469.5	(459.0–480.4)	80.0	(74.1–85.9)	666.4	(617.0–717.4)
3. 3.35–6.67	465.8	(457.8–473.8)	84.2	(80.1–88.3)	699.7	(664.9–735.5)
4. ≥6.68	459.2	(451.3–467.3)	87.1	(82.8–91.2)	693.5	(657.1–731.0)

a Adjusted for age, energy reporting quality, fat mass index and accelerometer wear time.

b Adjusted for age, energy reporting quality and fat mass index.

c Significant test for trend: *P* < 0.05.

**Table 3:** Estimated means and 95% confidence intervals (CI) of physical activity outcomes by quartiles of dietary variables for girls, holding other covariates to their means.

	Sedentary behaviour^a^ (min/day)		Moderate-to-vigorous physical activity^a^ (min/day)		Activity counts per min^b^ (min/day)	
Mean	95% CI	Mean	95% CI	Mean	95% CI
Energy (kcal)						
1. ≤1482.07	476.5	(466.1–486.4)	66.1	(60.8–71.4)	602.4	(547.8–657.7)
2. 1482.08–1707.03	474.4	(467.2–481.2)	68.4	(64.8–71.8)	645.3	(609.1–683.1)
3. 1707.04–1931.99	472.7	(466.2–479.4)	66.6	(63.3–70.0)	603.3	(566.7–638.9)
4. ≥1932.00	469.9	(459.3–480.2)	66.5	(61.1–71.7)	610.3	(552.6–670.1)
En% fat						
1. ≤34.05	476.3	(469.8–482.7)	65.5	(62.3–68.7)	599.9	(565.3–633.9)
2. 34.06–37.02	472.6	(466.3–478.9)	66.2	(63.0–69.4)	606.4	(574.2–641.9)
3. 37.03–40.07	473.2	(466.7–479.4)	68.0	(64.8–71.3)	627.3	(593.5–660.8)
4. ≥40.08	470.8	(464.2–477.2)	67.6	(64.2–70.9)	623.9	(589.6–656.8)
En% saturated fat						
1. ≤12.19	477.7	(471.5–484.6)	64.7	(61.6–68.0)	587.9	(554.8–623.4)
2. 12.20–13.69	472.6	(466.3–478.9)	67.7	(64.6–70.8)	623.0	(591.0–657.9)
3. 13.70–15.44	469.3	(463.2–475.7)	67.9	(64.7–71.3)	621.4	(586.7–657.1)
4. ≥15.45	473.5	(467.1–479.8)	67.0	(63.8–70.4)	625.5	(592.3–659.8)
En% protein						
1. ≤12.59	468.9	(462.3–475.2)	68.4	(65.2–71.8)	623.2	(589.3–657.6)
2. 12.60–14.06	472.1	(465.6–478.7)	67.7	(64.6–70.8)	623.9	(589.8–658.1)
3. 14.07–15.54	476.7	(470.3–483.2)	65.8	(62.5–68.9)	609.9	(577.0–644.8)
4. ≥15.55	475.7	(469.3–482.4)	65.4	(62.2–68.6)	602.7	(567.4–636.4)
En% carbohydrates						
1. ≤45.59	469.8	(463.8–476.3)	67.2	(64.1–70.4)	618.1	(586.7–651.9)
2. 45.60–48.74	475.3	(468.8–481.8)	67.7	(64.5–71.0)	632.7	(597.3–665.6)
3. 48.75–51.79	472.3	(465.9–478.7)	67.8	(64.7–71.1)	610.4	(577.4–644.9)
4. ≥51.80	475.8	(469.4–482.5)	64.6	(61.4–68.2)	599.4	(567.1–632.9)
Energy density (kJ/g)						
1. ≤5.28	475.8	(469.6–481.9)	66.1	(62.8–69.3)	616.0	(583.4–649.8)
2. 5.29–6.16	470.1	(463.8–476.7)	69.0	(65.7–72.4)	622.3	(588.5–657.2)
3. 6.17–7.23	475.9	(469.5–482.4)	65.3	(62.1–68.4)	605.1	(571.6–639.5)
4. ≥7.24	470.9	(464.3–477.7)	67.1	(63.8–70.4)	615.2	(581.1–649.8)
Fruit and vegetable (g/MJ)						
1. ≤15.97	472.8	(466.4–478.8)	66.1	(62.8–69.4)	609.1	(575.2–641.0)
2. 15.98–24.87	473.6	(467.3–480.2)	67.3	(63.9–70.6)	619.3	(586.2–652.4)
3. 24.88–35.87	475.6	(469.1–482.1)	65.8	(62.4–69.1)	600.7	(568.1–633.8)
4. ≥35.88	471.5	(465.1–478.5)	68.0	(65.0–71.5)	631.4	(600.4–665.7)
Fruit juices (g/MJ)						
1. ≤0	471.2	(464.6–477.0)	67.5	(64.3–70.5)	626.8	(594.2–659.8)
2. 0.01–14.23	476.5	(469.7–483.7)	65.5	(62.2–69.1)	608.0	(572.8–643.2)
3. 14.24–30.35	469.8	(462.9–476.2)	68.6	(65.5–71.7)	627.2	(593.7–661.8)
4. ≥30.36	475.5	(469.2–481.6)	65.6	(62.4–68.8)	596.7	(561.2–630.4)
Fizzy drinks (g/MJ)						
1. ≤0	474.5	(469.6–479.4)	66.6	(64.3–69.0)	613.7	(586.4–640.3)
2. 0.01–15.14	472.5	(465.9–479.0)	66.6	(63.4–70.0)	604.9	(571.8–639.9)
3. ≥15.15	471.9	(465.3–478.3)	67.5	(64.4–70.7)	627.1	(595.0–661.5)
Squash (g/MJ)						
1. ≤0	474.1	(468.1–480.2)	66.8	(63.9–69.7)	612.6	(580.0–645.3)
2. 0.01–13.87	468.5	(461.5–475.6)	69.3	(65.8–72.6)	617.3	(581.0–655.5)
3. 13.88–34.26	476.1	(469.7–482.7)	63.9	(60.7–67.2)	594.0	(561.7–630.4)
4. ≥34.27	473.9	(467.0–480.3)	67.9	(64.4–71.0)	634.1	(599.8–667.3)
Savoury snacks (g/MJ)						
1. ≤0.78	474.6	(468.4–481.0)	67.1	(63.9–70.2)	615.0	(580.2–647.7)
2. 0.79–1.60	474.3	(467.8–481.2)	64.9	(61.6–67.9)	595.5	(562.5–629.1)
3. 1.61–2.74	472.6	(466.3–479.5)	66.8	(63.6–70.0)	613.0	(578.4–646.3)
4. ≥2.75	471.4	(465.0–477.9)	68.7	(65.5–71.8)	637.1	(602.2–669.9)
Sweet snacks (g/MJ)						
1. ≤5.90	474.5	(468.1–481.0)	67.9	(64.5–71.2)	621.5	(586.2–655.9)
2. 5.91–9.76	473.3	(466.8–479.9)	65.2	(62.0–68.4)	598.7	(562.6–632.1)
3. 9.77–14.45	470.9	(464.5–477.3)	67.9	(64.8–71.0)	629.2	(595.7–664.4)
4. ≥14.46	474.8	(468.3–481.2)	66.4	(63.2–69.6)	610.4	(575.8–644.3)
Sweets/chocolate (g/MJ)						
1. ≤1.08	473.4	(467.4–479.8)	66.0	(63.0–69.3)	619.2	(584.4–652.4)
2. 1.09–2.35	473.9	(467.8–480.3)	66.7	(63.5–69.8)	616.2	(583.0–649.2)
3. 2.36–4.14	474.3	(467.8–480.8)	67.2	(64.0–70.5)	611.3	(575.7–645.8)
4. ≥4.15	471.2	(464.5–477.7)	67.5	(64.4–70.8)	613.4	(577.0–647.4)
Fries (g/MJ)						
1. ≤0	475.6	(469.8–481.2)	65.0	(62.1–67.9)	602.4	(572.3–634.2)
2. 0.01–3.81	472.0	(464.4–479.4)	69.5	(65.6–73.5)	645.9	(606.0–686.8)
3. 3.82–6.98	475.6	(469.2–482.0)	65.9	(62.7–69.0)	604.7	(572.0–638.9)
4. ≥6.99	468.8	(462.4–475.3)	68.6	(65.4–71.8)	622.0	(587.7–655.8)

a Adjusted for age, energy reporting quality, FMI and accelerometer wear time.

b Adjusted for age, energy reporting quality, FMI.
